# Supplementary material for: Provider-identified barriers to performance at seven Nigerian accident & emergency units: A cross-sectional study
Source: PLOS Glob Public Health. 2023 May 22;3(5):e0001909. doi: 10.1371/journal.pgph.0001909 (PMC10202278; doi:10.1371/journal.pgph.0001909)
Supplement: S1 Appendix — (PDF) [file pgph.0001909.s001.pdf]

Assessment of Emergency Care Services in Nigeria (AFEM Tool)

Facility Characteristics

Date and Time at Start of Interview

yyyy-mm-dd ____________ hh:mm ____________

Country

____

Name of Hospital you work in

____

City and State where the Hospital is located

____

Name of person filing out this questionnaire

____

When did you start working in this hospital?

yyyy-mm-dd ____________

Role in Facility

- ○ National Youth Service Copper (Youth Copper)
- ○ Medical Officer (Working Full-TIme in the A&E)
- ○ Medical Officer (Working Part-Time in the A&E)
- ○ Registrar
- ○ Senior Registrar
- ○ Consultant
- ○ Nurse
- ○ Other

If 'Other' please specify role

How many months in a given year do you work in the A&E?

If 'Registrar', 'Senior Registrar' or 'Consultant' please specify the department

- Internal Medicine
- Surgery
- Obstetrics and Gynecology
- Other

If Nurse, how many months in a given year do you work in the A&E?

If 'Other' please state the department

In a given year, how many months do you spend working in the A&E?

In a given year, how many months do you spend working in the Trauma unit (If there is one in your center)

Is there a Special Trauma center in your Hospital?

- Yes
- No

In any given day, for how many hours is the A&E unit open?

How many days a week is the A&E Unit open?

Is payment usually required before a patient is attended to?

- ◯ Yes
- ◯ No

Are patients required to purchase anything (like medications, or cotton wool, or other supplies) before they are attended to in the A&E?

- ◯ Yes
- ◯ No

Do you have any comments on required payment, or required supplies?

____

A. RESPIRATORY FAILURE

» I. Obstructed Airway

Use of manual maneuvers (e.g., jaw thrust, chin lift)	Generally not done	Sometimes done	Always done
**Are manual maneuvers (e.g., jaw thrust, chin lift) done for obstructed airway in the emergency unit?**	◯	◯	◯

Why are manual maneuvers (e.g., jaw thrust, chin lift) 'Somewhat done' or 'Generally not done' in the emergency unit?

- ☐ Infrastructure
- ☐ Absent Equipment
- ☐ Broken Equipment
- ☐ Inadequate Training
- ☐ Inadequate Personnel to do this
- ☐ Fees to be paid by patient
- ☐ No Indication
- ☐ Laws in the hospital prohibiting performance of this function
- ☐ Other reasons

Please state 'other' reason(s)

____

**Relief of obstruction (abdominal thrusts if conscious, CPR if unconscious, chest thrusts and back blows for infant)**	Generally not done	Sometimes done	Always done
**Are these Relief of obstruction maneuvers done in the emergency unit?**	○	○	○

Why are these Relief of obstruction maneuvers 'Somewhat done' or 'Generally not done' in the emergency unit?

- ☐ Infrastructure
- ☐ Absent Equipment
- ☐ Broken Equipment
- ☐ Inadequate Training
- ☐ Inadequate Personnel to do this
- ☐ Fees to be paid by patient
- ☐ No Indication
- ☐ Laws in the hospital prohibiting performance of this function
- ☐ Other reasons

Please state 'other' reason(s)

**Use of suction**	Generally not used	Sometimes used	Always Used
**Are suctions used in the emergency unit?**	○	○	○

Why are suctions 'sometimes used' or 'Generally not used' in the emergency unit?

- ☐ Infrastructure
- ☐ Absent Equipment
- ☐ Broken Equipment
- ☐ Inadequate Training
- ☐ Inadequate Personnel to do this
- ☐ Fees to be paid by patient
- ☐ No Indication
- ☐ Laws in the hospital prohibiting performance of this function
- ☐ Other reasons

Please state 'other' reason(s)

**Creation of surgical airway or cricothyrotomy**	Generally not created	Sometimes created	Always created
**Are surgical airways created in the emergency unit?**	○	○	○

Why are surgical airways (e.g tracheotomy or cricothyrotomy) 'Sometimes created ' or 'Generally not created' in the emergency unit?

- ☐ Infrastructure
- ☐ Absent Equipment
- ☐ Broken Equipment
- ☐ Inadequate Training
- ☐ Inadequate Personnel to do this
- ☐ Fees to be paid by patient
- ☐ No Indication
- ☐ Laws in the hospital prohibiting performance of this function
- ☐ Other reasons

Please state 'other' reason(s)

» » II. Respiratory Distress

**Rescue Breathing (Mouth-to-Mouth Resuscitation)**	Generally not done	Sometimes done	Always done
**Is Rescue Breathing (Mouth-to-Mouth Resuscitation) done in the emergency unit?**	○	○	○

Why is Rescue Breathing (Mouth-to-Mouth Resuscitation) 'sometimes done' or 'Generally not done' in the emergency unit?

- [ ] Infrastructure
- [ ] Absent Equipment
- [ ] Broken Equipment
- [ ] Inadequate Training
- [ ] Inadequate Personnel to do this
- [ ] Fees to be paid by patient
- [ ] No Indication
- [ ] Laws in the hospital prohibiting performance of this function
- [ ] Other reasons

Please state 'other' reasond(s)

____

**Three-way dressing (For Pneumothorax)**	Generally not done	Sometimes done	Always done
**In the setting of a pneumothorax, is a three-way dressing done in the emergency unit?**	○	○	○

Why is Three-way dressing (For Pneumothorax) 'sometimes done' or 'Generally not done' in the emergency unit?

- [ ] Infrastructure
- [ ] Absent Equipment
- [ ] Broken Equipment
- [ ] Inadequate Training
- [ ] Inadequate Personnel to do this
- [ ] Fees to be paid by patient
- [ ] No Indication
- [ ] Laws in the hospital prohibiting performance of this function
- [ ] Other reasons

Please state 'other' reason(s)

____

**Use of oro- or naso-pharyngeal airway device**	Generally not used	Sometimes used	Always used
**Are oro- or naso-pharyngeal airway devices used in the emergency unit?**	○	○	○

Why are oro- or naso-pharyngeal airway devices 'sometimes used' or 'Generally not used' in the emergency unit?

- [ ] Infrastructure
- [ ] Absent Equipment
- [ ] Broken Equipment
- [ ] Inadequate Training
- [ ] Inadequate Personnel to do this
- [ ] Fees to be paid by patient
- [ ] No Indication
- [ ] Laws in the hospital prohibiting performance of this function
- [ ] Other reasons

Please state 'other' reason(s)

____

**Bag-valve-mask ventilation**	Generally not used	Sometimes used	Always used
**Is Bag-valve-mask ventilation used in the emergency unit?**	○	○	○

Why is Bag-Valve-Mask Ventilation 'Sometimes used ' or 'Generally not used' in the emergency unit?

- [ ] Infrastructure
- [ ] Absent Equipment
- [ ] Broken Equipment
- [ ] Inadequate Training
- [ ] Inadequate Personnel to do this
- [ ] Fees to be paid by patient
- [ ] No Indication
- [ ] Laws in the hospital prohibiting performance of this function
- [ ] Other reasons

Please state 'other' reason(s)

**Use of supraglottic device (e.g., LMA)**	Generally not used	Sometimes used	Always used
**Are supraglottic devices (e.g., LMA) used in the emergency unit?**	○	○	○

Why are supraglottic devices (e.g., LMA) 'sometimes used' or 'Generally not used' in the emergency unit?

- ☐ Infrastructure
- ☐ Absent Equipment
- ☐ Broken Equipment
- ☐ Inadequate Training
- ☐ Inadequate Personnel to do this
- ☐ Fees to be paid by patient
- ☐ No Indication
- ☐ Laws in the hospital prohibiting performance of this function
- ☐ Other reasons

Please state 'other' reason(s)

**Administration of bronchodilator, adrenaline or steroids for reactive airway disease**	Generally not administered	Sometimes administered	Always Administered
**Are bronchodilators administered for reactive airway diseases in the emergency unit?**	○	○	○

Why are bronchodilators, adrenaline or steroids 'sometimes administered' or 'Generally not administered' for reactive airway disease?

- [ ] Infrastructure
- [ ] Absent Equipment
- [ ] Broken Equipment
- [ ] Inadequate Training
- [ ] Inadequate Personnel to do this
- [ ] Fees to be paid by patient
- [ ] No Indication
- [ ] Laws in the hospital prohibiting performance of this function
- [ ] Other reasons

Please state 'other' reason(s)

____

Administration of oxygen in the emergency unit	Generally not administered	Sometimes administered	Always Administered
Is oxygen administered in the emergency unit?	○	○	○

Why is Oxygen 'Sometimes administered' or 'Generally not administered' in the emergency unit?

- [ ] Infrastructure
- [ ] Absent Equipment
- [ ] Broken Equipment
- [ ] Inadequate Training
- [ ] Inadequate Personnel to do this
- [ ] Fees to be paid by patient
- [ ] No Indication
- [ ] Laws in the hospital prohibiting performance of this function
- [ ] Other reasons

Please state 'other' reason(s)

____

**Endotracheal intubation**	Generally not done	Sometimes done	Always done
**Are Endotracheal intubations done in the emergency unit?**	○	○	○

Why are Endotracheal intubations 'Sometimes done ' or 'Generally not done' in the emergency unit?

- ☐ Infrastructure
- ☐ Absent Equipment
- ☐ Broken Equipment
- ☐ Inadequate Training
- ☐ Inadequate Personnel to do this
- ☐ Fees to be paid by patient
- ☐ No Indication
- ☐ Laws in the hospital prohibiting performance of this function
- ☐ Other reasons

Please state 'other' reason(s)

____

**Creation of surgical airway /cricothyrotomy**	Generally not created	Sometimes created	Always created
**Are surgical airways created in the emergency unit?**	○	○	○

Why are surgical airways (e.g tracheotomy or cricothyrotomy) 'Sometimes created ' or 'Generally not created' in the emergency unit?

- ☐ Infrastructure
- ☐ Absent Equipment
- ☐ Broken Equipment
- ☐ Inadequate Training
- ☐ Inadequate Personnel to do this
- ☐ Fees to be paid by patient
- ☐ No Indication
- ☐ Laws in the hospital prohibiting performance of this function
- ☐ Other reasons

Please state 'other' reason(s)

**Non-invasive mechanical ventilation (BiPAP, CPAP) in the emergency unit**	Generally not used	Sometimes used	Always used
**Is non-invasive mechanical ventilation (BiPAP, CPAP) used in the emergency unit?**	○	○	○

Why is Non-invasive mechanical ventilation (BiPAP, CPAP) 'Sometimes used ' or 'Generally not used' in the emergency unit?

- [ ] Infrastructure
- [ ] Absent Equipment
- [ ] Broken Equipment
- [ ] Inadequate Training
- [ ] Inadequate Personnel to do this
- [ ] Fees to be paid by patient
- [ ] No Indication
- [ ] Laws in the hospital prohibiting performance of this function
- [ ] Other reasons

Please state 'other' reason(s)

**Invasive mechanical ventilation in the emergency unit**	Generally not used	Sometimes used	Always Used
**Is Invasive mechanical ventilation used in the emergency unit**	○	○	○

Why is invasive mechanical ventilation 'Sometimes used' or 'Generally not used' in the emergency unit?

- [ ] Infrastructure
- [ ] Absent Equipment
- [ ] Broken Equipment
- [ ] Inadequate Training
- [ ] Inadequate Personnel to do this
- [ ] Fees to be paid by patient
- [ ] No Indication
- [ ] Laws in the hospital prohibiting performance of this function
- [ ] Other reasons

Please state 'other' reason(s)

____

Do you have any comments concerning any of the above questions on Respiratory Failure?

____

SHOCK

» I. Haemorrhagic Shock

External control of hemorrhage	Generally not used	Sometimes used	Always used
**Are techniques for external control of bleeding (E.g Direct pressure, pressure bandage, pressure points) used in the emergency unit?**	○	○	○

Why are techniques for external control of bleeding 'sometimes used' or 'generally not used' in the emergency unit?

- [ ] Infrastructure
- [ ] Absent Equipment
- [ ] Broken Equipment
- [ ] Inadequate Training
- [ ] Inadequate Personnel to do this
- [ ] Fees to be paid by patient
- [ ] No Indication
- [ ] Laws in the hospital prohibiting performance of this function
- [ ] Other reasons

Please state 'other' reason(s)

**Tourniquet placement**	Generally not placed	Sometimes placed	Always used
**Are tourniquets placed to control bleeding in the emergency unit?**	○	○	○

Why are tourniquets 'sometimes placed' or 'generally not placed' in the emergency unit?

- [ ] Infrastructure
- [ ] Absent Equipment
- [ ] Broken Equipment
- [ ] Inadequate Training
- [ ] Inadequate Personnel to do this
- [ ] Fees to be paid by patient
- [ ] No Indication
- [ ] Laws in the hospital prohibiting performance of this function
- [ ] Other reasons

Please state 'other' reason(s)

**Pelvic binding/wrapping**	Generally not placed	Sometimes placed	Always placed

Are pelvic binders placed to control bleeding in the emergency unit? ○ ○ ○

Why are pelvic binders 'sometimes placed' or 'generally not placed' to control bleeding in the emergency unit?

- ☐ Infrastructure
- ☐ Absent Equipment
- ☐ Broken Equipment
- ☐ Inadequate Training
- ☐ Inadequate Personnel to do this
- ☐ Fees to be paid by patient
- ☐ No Indication
- ☐ Laws in the hospital prohibiting performance of this function
- ☐ Other reasons

Please state 'other' reason(s)

____

Wound packing and/or suture placement to control bleeding	Generally not placed	Sometimes placed	Always placed
**Are wounds packed and/or sutures placed to control bleeding in the emergency unit?**	○	○	○

Why are wound packings and/or sutures 'sometimes placed' or 'generally not placed' in the emergency unit?

- ☐ Infrastructure
- ☐ Absent Equipment
- ☐ Broken Equipment
- ☐ Inadequate Training
- ☐ Inadequate Personnel to do this
- ☐ Fees to be paid by patient
- ☐ No Indication
- ☐ Laws in the hospital prohibiting performance of this function
- ☐ Other reasons

Please state 'other' reason(s)

**Peripheral IV placement**	Generally not Placed	Sometimes Placed	Always Placed
**Are Peripheral IVs placed in the emergency unit?**	○	○	○

Why are Peripheral IVs 'sometimes placed' or 'generally not placed' in the emergency unit?

- ☐ Infrastructure
- ☐ Absent Equipment
- ☐ Broken Equipment
- ☐ Inadequate Training
- ☐ Inadequate Personnel to do this
- ☐ Fees to be paid by patient
- ☐ No Indication
- ☐ Laws in the hospital prohibiting performance of this function
- ☐ Other reasons

Please state 'other' reason(s)

**Intraosseous access**	Generally not obtained	Sometimes obtained	Always obtained
**Is Intraosseous access obtained in the emergency unit?**	○	○	○

Why is Intraosseous access 'sometimes obtained' or 'generally not obtained' in the emergency unit?

- [ ] Infrastructure
- [ ] Absent Equipment
- [ ] Broken Equipment
- [ ] Inadequate Training
- [ ] Inadequate Personnel to do this
- [ ] Fees to be paid by patient
- [ ] No Indication
- [ ] Laws in the hospital prohibiting performance of this function
- [ ] Other reasons

Please state 'other' reason(s)

**Venous cutdown**	Generally not done	Sometimes done	Always done
**Are Venous cutdowns done in the emergency unit?**	○	○	○

Why are Venous cutdowns 'sometimes done' or 'generally not done' in the emergency unit?

- [ ] Infrastructure
- [ ] Absent Equipment
- [ ] Broken Equipment
- [ ] Inadequate Training
- [ ] Inadequate Personnel to do this
- [ ] Fees to be paid by patient
- [ ] No Indication
- [ ] Laws in the hospital prohibiting performance of this function
- [ ] Other reasons

Please state 'other' reason(s)

**Safe transfusion (e.g., including screened blood, maintenance of sterility, monitoring)**	Generally not followed	Sometimes followed	Always followed

**Are safe transfusion protocols (including screened blood, maintenance of sterility, monitoring) followed in the emergency unit?**	○	○	○

Why are safe transfusion protocols (including screened blood, maintenance of sterility, monitoring) 'sometimes followed' or 'generally not followed' to control bleeding in the emergency unit?

- ☐ Infrastructure
- ☐ Absent Equipment
- ☐ Broken Equipment
- ☐ Inadequate Training
- ☐ Inadequate Personnel to do this
- ☐ Fees to be paid by patient
- ☐ No Indication
- ☐ Laws in the hospital prohibiting performance of this function
- ☐ Other reasons

Please state 'other' reason(s)

____

**Central venous line placement**	Generally not placed	Sometimes placed	Always Placed
**Are central venous lines placed in the emergency unit?**	○	○	○

Why are central venous lines 'sometimes placed ' or 'generally not placed' in the emergency unit?

- ☐ Infrastructure
- ☐ Absent Equipment
- ☐ Broken Equipment
- ☐ Inadequate Training
- ☐ Inadequate Personnel to do this
- ☐ Fees to be paid by patient
- ☐ No Indication
- ☐ Laws in the hospital prohibiting performance of this function
- ☐ Other reasons

Please state 'other' reason(s)

» III. OTHER SHOCK

**ECG with interpretation**	Generally not done and interpreted	Sometimes done and interpreted	Always done and interpreted
**Are ECGs done and interpreted in the emergency unit?**	○	○	○

Why are ECGs 'sometimes done and interpreted' or 'generally not done and interpreted' in the emergency unit?

- ☐ Infrastructure
- ☐ Absent Equipment
- ☐ Broken Equipment
- ☐ Inadequate Training
- ☐ Inadequate Personnel to do this
- ☐ Fees to be paid by patient
- ☐ No Indication
- ☐ Laws in the hospital prohibiting performance of this function
- ☐ Other reasons

Please state 'other' reason(s)

**External defibrillation and/or cardioversion**	Generally not done	Sometimes done	Always done
**Is external defibrillation and/or cardioversion done in the emergency unit?**	○	○	○

Why is External defibrillation and/or cardioversion 'sometimes done' or 'generally not done' in the emergency unit?

- [ ] Infrastructure
- [ ] Absent Equipment
- [ ] Broken Equipment
- [ ] Inadequate Training
- [ ] Inadequate Personnel to do this
- [ ] Fees to be paid by patient
- [ ] No Indication
- [ ] Laws in the hospital prohibiting performance of this function
- [ ] Other reasons

Please state 'other' reason(s)

**Needle decompression of tension pneumothorax**	Generally not done	Sometimes done	Always done
**Is Needle decompression done for tension pneumothorax in the emergency unit?**	○	○	○

Why is External defibrillation and/or cardioversion 'sometimes done' or 'generally not done' in the emergency unit?

- [ ] Infrastructure
- [ ] Absent Equipment
- [ ] Broken Equipment
- [ ] Inadequate Training
- [ ] Inadequate Personnel to do this
- [ ] Fees to be paid by patient
- [ ] No Indication
- [ ] Laws in the hospital prohibiting performance of this function
- [ ] Other reasons

Please state 'other' reason(s)

**Adrenaline/Epinephrine administration for anaphylactic shock**	Generally not administered	Sometimes administered	Always administered
**Is Adrenaline/Epinephrine administered in the emergency unit?**	○	○	○

Why is Adrenaline/Epinephrine 'sometimes administered ' or 'generally not administered' in the emergency unit?

- [ ] Infrastructure
- [ ] Absent Equipment
- [ ] Broken Equipment
- [ ] Inadequate Training
- [ ] Inadequate Personnel to do this
- [ ] Fees to be paid by patient
- [ ] No Indication
- [ ] Laws in the hospital prohibiting performance of this function
- [ ] Other reasons

Please state 'other' reason(s)

____

**IV vasopressor administration**	Generally not administered	Sometimes administered	Always administered
**Are IV vasopressors administered in the emergency unit?**	○	○	○

Why are IV vasopressors 'sometimes administered' or 'generally not administered' in the emergency unit?

- [ ] Infrastructure
- [ ] Absent Equipment
- [ ] Broken Equipment
- [ ] Inadequate Training
- [ ] Inadequate Personnel to do this
- [ ] Fees to be paid by patient
- [ ] No Indication
- [ ] Laws in the hospital prohibiting performance of this function
- [ ] Other reasons

Please state 'other' reason(s)

____

**Thrombolytic administration for Myocardial Ischemia**	Generally not administered	Sometimes administered	Always administered
**Are thrombolytics administered for myocardial ischemia in the emergency unit?**	○	○	○

Why are thrombolytics 'sometimes administered' or 'generally not administered' for myocardial infarction in the emergency unit?

- [ ] Infrastructure
- [ ] Absent Equipment
- [ ] Broken Equipment
- [ ] Inadequate Training
- [ ] Inadequate Personnel to do this
- [ ] Fees to be paid by patient
- [ ] No Indication
- [ ] Laws in the hospital prohibiting performance of this function
- [ ] Other reasons

Please state 'other' reason(s)

____

**Pericardiocentesis**	Generally not performed	Sometimes performed	Always Performed
**Is Pericardiocentesis performed in the emergency unit?**	○	○	○

Why is Pericardiocentesis 'sometimes performed' or 'generally not performed' in the emergency unit?

- ☐ Infrastructure
- ☐ Absent Equipment
- ☐ Broken Equipment
- ☐ Inadequate Training
- ☐ Inadequate Personnel to do this
- ☐ Fees to be paid by patient
- ☐ No Indication
- ☐ Laws in the hospital prohibiting performance of this function
- ☐ Other reasons

Please state 'other' reason(s)

____

» III. Severe Sepsis/Septic Shock

**Isotonic IV fluid administration**	Generally not administered	Sometimes administere d	Always administered
**Are Isotonic IV fluids administered in the emergency unit?**	○	○	○

Why are Isotonic IV fluids 'sometimes administered' or 'generally not administered' in the emergency unit?

- [ ] Infrastructure
- [ ] Absent Equipment
- [ ] Broken Equipment
- [ ] Inadequate Training
- [ ] Inadequate Personnel to do this
- [ ] Fees to be paid by patient
- [ ] No Indication
- [ ] Laws in the hospital prohibiting performance of this function
- [ ] Other reasons

Please state 'other' reason(s)

____

**IV antibiotic administration**	Generally not administered	Sometimes administere d	Always administered
**Are IV antibiotics and /or antimalarials administered in the emergency unit?**	○	○	○

Why are IV antibiotics and/or antimalarials 'sometimes administered' or 'generally not administered' in the emergency unit?

- [ ] Infrastructure
- [ ] Absent Equipment
- [ ] Broken Equipment
- [ ] Inadequate Training
- [ ] Inadequate Personnel to do this
- [ ] Fees to be paid by patient
- [ ] No Indication
- [ ] Laws in the hospital prohibiting performance of this function
- [ ] Other reasons

Please state 'other' reason(s)

____

Do you have any comments concerning any of the above questions on Shock?

ALTERED MENTAL STATUS

» I. Unconscious patient

In the setting of Altered Mental Status are blood pressure and oxygen saturation monitored and Nasogastric tube placed to reduce aspiration?	Generally not available	Sometimes available	Always
When a patient comes in with altered mental status, are the measures mentioned above, put in place to protect patients from secondary injury in the emergency unit?	○	○	○

Why are the measures (listed above) to protect patients from secondary injury in the case of a altered mental status 'sometimes available' or 'generally not available' in the emergency unit?

- ☐ Infrastructure
- ☐ Absent Equipment
- ☐ Broken Equipment
- ☐ Inadequate Training
- ☐ Inadequate Personnel to do this
- ☐ Fees to be paid by patient
- ☐ No Indication
- ☐ Laws in the hospital prohibiting performance of this function
- ☐ Other reasons

Please state 'other' reason(s)

Check and/or administration of Glucose for hypoglycemia	Generally not administered	Sometimes administered	Always administered
Is blood glucose check and /or administered to hypoglycemic patients in the emergency unit?	○	○	○

Why is glucose 'sometimes administered' or 'generally not administered' to hypoglycemic patients in the emergency unit?

- [ ] Infrastructure
- [ ] Absent Equipment
- [ ] Broken Equipment
- [ ] Inadequate Training
- [ ] Inadequate Personnel to do this
- [ ] Fees to be paid by patient
- [ ] No Indication
- [ ] Laws in the hospital prohibiting performance of this function
- [ ] Other reasons

Please state 'other' reason(s)

____

**Insulin administration for hyperglycemia**	Generally not done	Sometimes done	Always done
**In the setting of hyperglycemia, is Insulin administered in the emergency unit?**	○	○	○

Why is Insulin 'sometimes administered' or 'generally not administered' in the emergency unit for patients with hyperglycemia?

- [ ] Infrastructure
- [ ] Absent Equipment
- [ ] Broken Equipment
- [ ] Inadequate Training
- [ ] Inadequate Personnel to do this
- [ ] Fees to be paid by patient
- [ ] No Indication
- [ ] Laws in the hospital prohibiting performance of this function
- [ ] Other reasons

Please state 'other' reason(s)

____

**Head CT**	Generally not done	Sometimes done	Always done
**In the setting of altered mental status, is Head CT done in the emergency unit?**	○	○	○

Why are lumbar punctures 'sometimes done' or 'generally not done' in the emergency unit?

- ☐ Infrastructure
- ☐ Absent Equipment
- ☐ Broken Equipment
- ☐ Inadequate Training
- ☐ Inadequate Personnel to do this
- ☐ Fees to be paid by patient
- ☐ No Indication
- ☐ Laws in the hospital prohibiting performance of this function
- ☐ Other reasons

Please state 'other' reason(s)

**Lumbar puncture**	Generally not done	Sometimes done	Always done
**Are lumbar punctures done in the emergency unit?**	○	○	○

Why are lumbar punctures 'sometimes done' or 'generally not done' in the emergency unit?

- ☐ Infrastructure
- ☐ Absent Equipment
- ☐ Broken Equipment
- ☐ Inadequate Training
- ☐ Inadequate Personnel to do this
- ☐ Fees to be paid by patient
- ☐ No Indication
- ☐ Laws in the hospital prohibiting performance of this function
- ☐ Other reasons

Please state 'other' reason(s)

» II. Seizure

Benzodiazepine administration	Generally not administered	Sometimes administered	Always administered
Are Benzodiazepines administered in the emergency unit?	○	○	○

In the case of seizures, why are benzodiazepines 'sometimes administered' or 'generally not administered' in the emergency unit?

- ☐ Infrastructure
- ☐ Absent Equipment
- ☐ Broken Equipment
- ☐ Inadequate Training
- ☐ Inadequate Personnel to do this
- ☐ Fees to be paid by patient
- ☐ No Indication
- ☐ Laws in the hospital prohibiting performance of this function
- ☐ Other reasons

Please state 'other' reason(s)

IV magnesium sulphate administration for pregnant women	Generally not done	Sometimes done	Always done
In the setting of eclampsia, is IV magnesium administered in the emergency unit?	○	○	○

Why is IV magnesium 'sometimes administered' or 'generally not administered' in the emergency unit for patients with eclampsia?

- [ ] Infrastructure
- [ ] Absent Equipment
- [ ] Broken Equipment
- [ ] Inadequate Training
- [ ] Inadequate Personnel to do this
- [ ] Fees to be paid by patient
- [ ] No Indication
- [ ] Laws in the hospital prohibiting performance of this function
- [ ] Other reasons

Please state 'other' reason(s)

____

**Relevant antidote administration for toxic exposure (eg, atropine, naloxone, anti-venin)**	Generally not administered	Sometimes administered	Always administered
**In the setting of toxic exposure, are relevant antidotes (eg, atropine, naloxone, anti-venin) administered in the emergency unit?**	○	○	○

In the setting of toxic exposure, why are relevant antidotes (eg, atropine, naloxone, anti-venin) 'sometimes administered' or 'generally not administered' in the emergency unit?

- [ ] Infrastructure
- [ ] Absent Equipment
- [ ] Broken Equipment
- [ ] Inadequate Training
- [ ] Inadequate Personnel to do this
- [ ] Fees to be paid by patient
- [ ] No Indication
- [ ] Laws in the hospital prohibiting performance of this function
- [ ] Other reasons

Please state 'other' reason(s)

» III. Other

**Mental status examination administration**	Generally not done	Sometimes done	Always done
**Are Mental status examinations done in the emergency unit?**	○	○	○

Why are Mental status examinations 'sometimes done' or 'generally not done' in the emergency unit?

- ☐ Infrastructure
- ☐ Absent Equipment
- ☐ Broken Equipment
- ☐ Inadequate Training
- ☐ Inadequate Personnel to do this
- ☐ Fees to be paid by patient
- ☐ No Indication
- ☐ Laws in the hospital prohibiting performance of this function
- ☐ Other reasons

Please state 'other' reason(s)

**Extreme temperature management (hyper- or hypothermia)**	Generally not managed	Sometimes managed	Always managed
**Are patients with extreme temperatures (hyper- or hypothermia) managed in the emergency unit?**	○	○	○

Why are patients with extreme temperatures (hyper- or hypothermia) 'sometimes managed' or 'generally not managed' in the emergency unit?

- [ ] Infrastructure
- [ ] Absent Equipment
- [ ] Broken Equipment
- [ ] Inadequate Training
- [ ] Inadequate Personnel to do this
- [ ] Fees to be paid by patient
- [ ] No Indication
- [ ] Laws in the hospital prohibiting performance of this function
- [ ] Other reasons

Please state 'other' reason(s)

____

Do you have any comments concerning any of the above questions on neurologic interventions?

____

SEVERE PAIN

» I. General Severe Pain

**Opiate analgesia administration**	Generally not administered	Sometimes administered	Always administered
**Are opiate analgesics administered in the emergency or trauma unit (if there is one at your hospital)?**	○	○	○

Why are opiate analgesics 'sometimes administered' or 'generally not administered' in the emergency unit?

- [ ] Infrastructure
- [ ] Absent Equipment
- [ ] Broken Equipment
- [ ] Inadequate Training
- [ ] Inadequate Personnel to do this
- [ ] Fees to be paid by patient
- [ ] No Indication
- [ ] Laws in the hospital prohibiting performance of this function
- [ ] Other reasons

Please state 'other' reason(s)

» II. Abdominal Pain

Urine Dipstick (urinalysis)	Generally not performed	Sometimes performed	Always performed
**Is Urine Dipstick (urinalysis) performed in the emergency unit?**	○	○	○

Why is Urine Dipstick (urinalysis) 'sometimes performed' or 'generally not performed' in the emergency unit?

- [ ] Infrastructure
- [ ] Absent Equipment
- [ ] Broken Equipment
- [ ] Inadequate Training
- [ ] Inadequate Personnel to do this
- [ ] Fees to be paid by patient
- [ ] No Indication
- [ ] Laws in the hospital prohibiting performance of this function
- [ ] Other reasons

Please state 'other' reason(s)

**HCG Testing**	Generally not performed	Sometimes performed	Always performed
**Is HCG Testing performed in the emergency unit?**	○	○	○

Why is HCG Testing 'sometimes performed' or 'generally not performed' in the emergency unit?

- ☐ Infrastructure
- ☐ Absent Equipment
- ☐ Broken Equipment
- ☐ Inadequate Training
- ☐ Inadequate Personnel to do this
- ☐ Fees to be paid by patient
- ☐ No Indication
- ☐ Laws in the hospital prohibiting performance of this function
- ☐ Other reasons

Please state 'other' reason(s)

**Administration of oral rehydration**	Generally not administered	Sometimes administered	Always administered
**Is oral rehydration administered in the emergency unit?**	○	○	○

Why is oral rehydration 'sometimes administered' or 'generally not administered' in the emergency unit?

- [ ] Infrastructure
- [ ] Absent Equipment
- [ ] Broken Equipment
- [ ] Inadequate Training
- [ ] Inadequate Personnel to do this
- [ ] Fees to be paid by patient
- [ ] No Indication
- [ ] Laws in the hospital prohibiting performance of this function
- [ ] Other reasons

Please state 'other' reason(s)

**Urinary catheter placement**	Generally not placed	Sometimes placed	Always Placed
**Are Urinary catheters placed in the emergency unit?**	○	○	○

Why are Urinary catheters 'sometimes placed ' or 'generally not placed' in the emergency unit?

- [ ] Infrastructure
- [ ] Absent Equipment
- [ ] Broken Equipment
- [ ] Inadequate Training
- [ ] Inadequate Personnel to do this
- [ ] Fees to be paid by patient
- [ ] No Indication
- [ ] Laws in the hospital prohibiting performance of this function
- [ ] Other reasons

Please state 'other' reason(s)

**Diagnostic and therapeutic paracentesis**	Generally not performed	Sometimes performed	Always performed

Are diagnostic and/or therapeutic paracenteses performed in the emergency unit? ◯ ◯ ◯

Why are diagnostic paracenteses 'sometimes performed' or 'generally not performed' in the emergency unit?

- ☐ Infrastructure
- ☐ Absent Equipment
- ☐ Broken Equipment
- ☐ Inadequate Training
- ☐ Inadequate Personnel to do this
- ☐ Fees to be paid by patient
- ☐ No Indication
- ☐ Laws in the hospital prohibiting performance of this function
- ☐ Other reasons

Please state 'other' reason(s)

Ultrasound (performance and interpretation)	Generally not done	Sometimes done	Always done
**Is ultrasound (performance and interpretation) done in the emergency unit?**	◯	◯	◯

Why is ultrasound 'sometimes used' or 'generally not used' in the emergency unit?

- ☐ Infrastructure
- ☐ Absent Equipment
- ☐ Broken Equipment
- ☐ Inadequate Training
- ☐ Inadequate Personnel to do this
- ☐ Fees to be paid by patient
- ☐ No Indication
- ☐ Laws in the hospital prohibiting performance of this function
- ☐ Other reasons

Please state 'other' reason(s)

» III. Chest Pain

**Aspirin administration for acute coronary syndrome**	Generally not administered	Sometimes administered	Always administered
**Is Aspirin administered for acute coronary syndrome in the emergency unit?**	○	○	○

Why is Aspirin 'sometimes administered' or 'generally not administered' for Ischemia in the emergency unit?

- ☐ Infrastructure
- ☐ Absent Equipment
- ☐ Broken Equipment
- ☐ Inadequate Training
- ☐ Inadequate Personnel to do this
- ☐ Fees to be paid by patient
- ☐ No Indication
- ☐ Laws in the hospital prohibiting performance of this function
- ☐ Other reasons

Please state 'other' reason(s)

**Chest X-Ray**	Generally not administered	Sometimes administered	Always administered
**Are Chest X-Rays done in the emergency unit?**	○	○	○

Why are Chest X-rays 'sometimes done' or 'generally not done' in the emergency unit?

- [ ] Infrastructure
- [ ] Absent Equipment
- [ ] Broken Equipment
- [ ] Inadequate Training
- [ ] Inadequate Personnel to do this
- [ ] Fees to be paid by patient
- [ ] No Indication
- [ ] Laws in the hospital prohibiting performance of this function
- [ ] Other reasons

Please state 'other' reason(s)

Do you have any comments concerning any of the above questions on Altered Mental Status?

TRAUMA INTERVENTIONS

» I. General Trauma

Trauma protocol implementation	Generally not done	Sometimes done	Always done
**Are trauma protocols implemented in the A&E?**	○	○	○

Why are cervical spine immobilizations 'sometimes done' or 'generally not done' in the emergency unit?

- [ ] Infrastructure
- [ ] Absent Equipment
- [ ] Broken Equipment
- [ ] Inadequate Training
- [ ] Inadequate Personnel to do this
- [ ] Fees to be paid by patient
- [ ] No Indication
- [ ] Laws in the hospital prohibiting performance of this function
- [ ] Other reasons

Please state 'other' reason(s)

**Initial wound care (irrigate with potable water or sterile solution, surgically close clean acute wounds, dress, infection control)**	Generally not done	Sometimes done	Always done
**Is initial wound care done in the emergency or trauma unit (if there is one at your hospital)?**	○	○	○

Why is initial wound care 'sometimes done' or 'generally not done' in the emergency unit?

- [ ] Infrastructure
- [ ] Absent Equipment
- [ ] Broken Equipment
- [ ] Inadequate Training
- [ ] Inadequate Personnel to do this
- [ ] Fees to be paid by patient
- [ ] No Indication
- [ ] Laws in the hospital prohibiting performance of this function
- [ ] Other reasons

Please state 'other' reason(s)

Fracture immobilization

	Generally not done	Sometimes done	Always done
**Are fracture immobilizations done in the emergency or trauma unit (if there is one at your hospital)?**	○	○	○

Why are fracture immobilizations 'sometimes done' or 'generally not done' in the emergency unit?

- ☐ Infrastructure
- ☐ Absent Equipment
- ☐ Broken Equipment
- ☐ Inadequate Training
- ☐ Inadequate Personnel to do this
- ☐ Fees to be paid by patient
- ☐ No Indication
- ☐ Laws in the hospital prohibiting performance of this function
- ☐ Other reasons

Please state 'other' reason(s)

____

Fracture Reduction

	Generally not done	Sometimes done	Always done
**Are fracture reductions done in the emergency or trauma unit (if there is one at your hospital)?**	○	○	○

Why are fracture reductions 'sometimes done' or 'generally not done' in the emergency unit?

- ☐ Infrastructure
- ☐ Absent Equipment
- ☐ Broken Equipment
- ☐ Inadequate Training
- ☐ Inadequate Personnel to do this
- ☐ Fees to be paid by patient
- ☐ No Indication
- ☐ Laws in the hospital prohibiting performance of this function
- ☐ Other reasons

Please state 'other' reason(s)

**Cervical spine immobilization**	Generally not done	Sometimes done	Always done
**Are cervical spine immobilizations done in the emergency or trauma unit (if there is one at your hospital)?**	○	○	○

Why are cervical spine immobilizations 'sometimes done' or 'generally not done' in the emergency unit?

- [ ] Infrastructure
- [ ] Absent Equipment
- [ ] Broken Equipment
- [ ] Inadequate Training
- [ ] Inadequate Personnel to do this
- [ ] Fees to be paid by patient
- [ ] No Indication
- [ ] Laws in the hospital prohibiting performance of this function
- [ ] Other reasons

Please state 'other' reason(s)

**Tetanus vaccination or IVIg as appropriate**	Generally not administered	Sometimes administered	Always administered
**Are Tetanus vaccinations or IVIg administered as appropriate in the emergency or trauma unit (if there is one at your hospital)?**	○	○	○

Why are tetanus vaccinations or IVIg 'sometimes administered' or 'generally not administered' as appropriate in the emergency unit?

- [ ] Infrastructure
- [ ] Absent Equipment
- [ ] Broken Equipment
- [ ] Inadequate Training
- [ ] Inadequate Personnel to do this
- [ ] Fees to be paid by patient
- [ ] No Indication
- [ ] Laws in the hospital prohibiting performance of this function
- [ ] Other reasons

Please state 'other' reason(s)

____

**Antibiotic administration for open fractures**	Generally not administered	Sometimes administered	Always administered
**Are antibiotics administered for open fractures in the emergency or trauma unit (if there is one at your hospital)?**	○	○	○

Why are antibiotics 'sometimes administered' or 'generally not administered' in the emergency unit?

- [ ] Infrastructure
- [ ] Absent Equipment
- [ ] Broken Equipment
- [ ] Inadequate Training
- [ ] Inadequate Personnel to do this
- [ ] Fees to be paid by patient
- [ ] No Indication
- [ ] Laws in the hospital prohibiting performance of this function
- [ ] Other reasons

Please state 'other' reason(s)

____

**Fasciotomy for compartment syndrome**	Generally not performed	Sometimes performed	Always performed
**In the setting of compartment syndrome, are fasciotomies or escharotomies performed in the emergency or trauma unit (if there is one at your hospital)?**	○	○	○

In the setting of compartment syndrome, why are fasciotomies 'sometimes done' or 'generally not done' in the emergency unit?

- [ ] Infrastructure
- [ ] Absent Equipment
- [ ] Broken Equipment
- [ ] Inadequate Training
- [ ] Inadequate Personnel to do this
- [ ] Fees to be paid by patient
- [ ] No Indication
- [ ] Laws in the hospital prohibiting performance of this function
- [ ] Other reasons

Please state 'other' reason(s)

**Rabies vaccination or IVIg as appropriate**	Generally not administered	Sometimes administered	Always administered
**Are Rabies vaccinations or IVIg administered as appropriate in the emergency or trauma unit (if there is one at your hospital)?**	○	○	○

Why are rabies vaccination or IVIg 'sometimes administered' or 'generally not administered' as appropriate in the emergency unit?

- [ ] Infrastructure
- [ ] Absent Equipment
- [ ] Broken Equipment
- [ ] Inadequate Training
- [ ] Inadequate Personnel to do this
- [ ] Fees to be paid by patient
- [ ] No Indication
- [ ] Laws in the hospital prohibiting performance of this function
- [ ] Other reasons

Please state 'other' reason(s)

**Access to general definitive surgical services**	Generally not done	Sometimes done	Always done
**Is there access to general definitive surgical services in the emergency or trauma unit at your hospital?**	○	○	○

Why is access to general definitive surgical services 'sometimes available' or 'generally not available' in the emergency unit?

- [ ] Infrastructure
- [ ] Absent Equipment
- [ ] Broken Equipment
- [ ] Inadequate Training
- [ ] Inadequate Personnel to do this
- [ ] Fees to be paid by patient
- [ ] No Indication
- [ ] Laws in the hospital prohibiting performance of this function
- [ ] Other reasons

Please state 'other' reason(s)

**Access to orthopaedic surgical services**	Generally not done	Sometimes done	Always done
**Is there access to orthopaedic surgical services in the emergency or trauma unit at your hospital?**	○	○	○

Why is access to orthopaedic surgical services 'sometimes available' or 'generally not available' in the emergency unit?

- ☐ Infrastructure
- ☐ Absent Equipment
- ☐ Broken Equipment
- ☐ Inadequate Training
- ☐ Inadequate Personnel to do this
- ☐ Fees to be paid by patient
- ☐ No Indication
- ☐ Laws in the hospital prohibiting performance of this function
- ☐ Other reasons

Please state 'other' reason(s)

**Access to neurosurgical services**	Generally not available	Sometimes available	Always available
**Is there access to neurosurgical services in the emergency or trauma unit at your hospital?**	○	○	○

Why is access to neurosurgical services 'sometimes available' or 'generally not available' in the emergency unit?

- ☐ Infrastructure
- ☐ Absent Equipment
- ☐ Broken Equipment
- ☐ Inadequate Training
- ☐ Inadequate Personnel to do this
- ☐ Fees to be paid by patient
- ☐ No Indication
- ☐ Laws in the hospital prohibiting performance of this function
- ☐ Other reasons

Please state 'other' reason(s)

**Placement of chest tube**	Generally not placed	Sometimes placed	Always placed
**Are chest tubes placed in the emergency unit?**	○	○	○

Why are Chest Tubes 'Sometimes placed ' or 'Generally not placed' in the emergency unit?

- [ ] Infrastructure
- [ ] Absent Equipment
- [ ] Broken Equipment
- [ ] Inadequate Training
- [ ] Inadequate Personnel to do this
- [ ] Fees to be paid by patient
- [ ] No Indication
- [ ] Laws in the hospital prohibiting performance of this function
- [ ] Other reasons

Please state 'other' reason(s)

**Thoracotomy in the emergency unit**	Generally not done	Sometimes done	Always done
**Are thoracotomies done in the emergency unit?**	○	○	○

Why are thoracotomies 'Sometimes done' or 'Generally not done' in the emergency unit?

- [ ] Infrastructure
- [ ] Absent Equipment
- [ ] Broken Equipment
- [ ] Inadequate Training
- [ ] Inadequate Personnel to do this
- [ ] Fees to be paid by patient
- [ ] No Indication
- [ ] Laws in the hospital prohibiting performance of this function
- [ ] Other reasons

Please state 'other' reason(s)

____

**Autotransfusion from chest tubes**	Generally not done	Sometimes done	Always done
**Are Autotransfusion from chest tubes done in the emergency unit?**	○	○	○

Why are Autotransfusions from chest tubes 'Sometimes done' or 'Generally not done' in the emergency unit?

- [ ] Infrastructure
- [ ] Absent Equipment
- [ ] Broken Equipment
- [ ] Inadequate Training
- [ ] Inadequate Personnel to do this
- [ ] Fees to be paid by patient
- [ ] No Indication
- [ ] Laws in the hospital prohibiting performance of this function
- [ ] Other reasons

Please state 'other' reason(s)

____

» II. Burns

**Escharatomy**	Generally not done	Sometimes done	Always done
**Are escharotomies done for burn patients in the emergency unit?**	○	○	○

Why are escharotomies 'Sometimes done' or 'Generally not done' in the emergency unit?

- ☐ Infrastructure
- ☐ Absent Equipment
- ☐ Broken Equipment
- ☐ Inadequate Training
- ☐ Inadequate Personnel to do this
- ☐ Fees to be paid by patient
- ☐ No Indication
- ☐ Laws in the hospital prohibiting performance of this function
- ☐ Other reasons

Please state 'other' reason(s)

____

**Cooling Care for burn patients**	Generally not done	Sometimes done	Always done
**Is Cooling care done for burn patients in the emergency unit?**	○	○	○

Why is cooling care 'Sometimes done' or 'Generally not done' in the emergency unit?

- ☐ Infrastructure
- ☐ Absent Equipment
- ☐ Broken Equipment
- ☐ Inadequate Training
- ☐ Inadequate Personnel to do this
- ☐ Fees to be paid by patient
- ☐ No Indication
- ☐ Laws in the hospital prohibiting performance of this function
- ☐ Other reasons

Please state 'other' reason(s)

Do you have any comments concerning any of the above questions on Trauma?

MATERNAL HEALTH

» Obstructed Labor

**Administration of uterotonic drugs (e.g IV oxytocin)**	Generally not given	Sometimes given	Always given
**Are uterotonic drugs (e.g IV oxytocin) given in the emergency unit?**	○	○	○

Why are uterotonic drugs (e.g., oxytocin) 'sometimes administered' or 'generally not administered' in the emergency unit?

- ☐ Infrastructure
- ☐ Absent Equipment
- ☐ Broken Equipment
- ☐ Inadequate Training
- ☐ Inadequate Personnel to do this
- ☐ Fees to be paid by patient
- ☐ No Indication
- ☐ Laws in the hospital prohibiting performance of this function
- ☐ Other reasons

Please state 'other' reason(s)

**Assisted vaginal delivery (e.g with vacuum or forceps)**	Generally not done	Sometimes done	Always done
**Are assisted vaginal deliveries (e.g with vacuum or forceps) done in the emergency unit?**	○	○	○

Why are uterotonic drugs (e.g., oxytocin) 'sometimes administered' or 'generally not administered' in the emergency unit?

- [ ] Infrastructure
- [ ] Absent Equipment
- [ ] Broken Equipment
- [ ] Inadequate Training
- [ ] Inadequate Personnel to do this
- [ ] Fees to be paid by patient
- [ ] No Indication
- [ ] Laws in the hospital prohibiting performance of this function
- [ ] Other reasons

Please state 'other' reason(s)

Access to surgical services (e.g. caesarean section)	Generally not available	Sometimes available	Always available
Is there access to emergency surgical services (e.g. caesarean section) in the emergency unit?	○	○	○

Why are emergency surgical services (e.g. caesarean section) 'sometimes available' or 'generally not available' in the emergency unit?

- [ ] Infrastructure
- [ ] Absent Equipment
- [ ] Broken Equipment
- [ ] Inadequate Training
- [ ] Inadequate Personnel to do this
- [ ] Fees to be paid by patient
- [ ] No Indication
- [ ] Laws in the hospital prohibiting performance of this function
- [ ] Other reasons

Please state 'other' reason(s)

**Neonatal (or newborn) resuscitation**	Generally not done	Sometimes done	Always done
**Are neonatal resuscitations done in the emergency unit?**	○	○	○

Why are neonatal resuscitations 'sometimes done' or 'generally not done' in the emergency unit?

- ☐ Infrastructure
- ☐ Absent Equipment
- ☐ Broken Equipment
- ☐ Inadequate Training
- ☐ Inadequate Personnel to do this
- ☐ Fees to be paid by patient
- ☐ No Indication
- ☐ Laws in the hospital prohibiting performance of this function
- ☐ Other reasons

Please state 'other' reason(s)

____

Do you have any comments concerning any of the above questions on Maternal Health?

____

CONCLUSION

We are done with this interview. Do you have any comments or feedback about the interview process or the questions asked?

____

Time at End of Interview

hh:mm
