# Supplementary material for: Provider-identified barriers to performance at seven Nigerian accident & emergency units: A cross-sectional study
Source: PLOS Glob Public Health. 2023 May 22;3(5):e0001909. doi: 10.1371/journal.pgph.0001909 (PMC10202278; doi:10.1371/journal.pgph.0001909)
Supplement: Attachment — Submitted filename: Barriers Reviewer Responses.docx [file pgph.0001909.s002.docx]

**Reviewer Responses - Barriers to Performance at Seven Nigerian Accident & Emergency Units**

1. *Please see these publications which caution against the ubiquitous and unreflective use of the term "LMIC”. At best, this term tends to over-generalize.*

Thank you for this feedback. We have changed our use of ‘LMIC” to ‘resource-variable’, reflecting current conventions in the language for global health research, and that many of the constraints seen around global medical care may also be seen in some high income contexts also. We have additionally cited literature that makes use of this language in the global health context.

See:

Missett RM, Kynes JM. Emphasis on Equity: Moving Global Health Education Forward. ASA Monit. 2021 Oct 1;85(10):33–4.

2. *Please clarify the process of randomization for the selection of included study sites.*

We now specify that randomization was performed using [www.random.org](http://www.random.org) in our methods section. We initially included this detail in our sister study (see response 9), and was not included here out of concern for redundancy and self-plagiarism, as well as mindfulness towards word count limits. All the same, we now include it per your recommendations.

3. *Please clarify what you mean by a “universal sampling technique”. It is not clear how you decided how many of each cadre of provider needed to be interviewed at each site. It’s unclear why there were more providers at some sites than others, and some providers were omitted entirely in some sites.*

Survey data was collected using universal and convenience sampling. We took a universal approach to physicians, interviewing all doctors identified using rotation schedules as frequent A&E staff, and who were identified by the head of each hospital’s A&E department as having worked in the A&E department within the three months preceding data collection. We also hoped to include perspectives from nurses for triangulation of responses provided by physicians, the population targeted in prior uses of the ECAT. In African contexts, nurses may be critically important to the provision of emergency care, with crucial roles in the identification and triage of emergency medical conditions and patients, as well as direct patient care, making their perspectives particularly valuable for this type of analysis. That this study was funded using a nominal trainee grant, limited out ability to take a universal approach to both cadres of provider, and we instead opted for a convenience sample approach for data collection from nurses. These nurses were non-rotating staff at each A&E, and were sought out by data collectors at the time of each site visit.  We have added our reasoning around our approach to nurses to the article, and cite studies that reflect their importance in African emergency medicine.

See:

Calvello E, Reynolds T, Hirshon JM, Buckle C, Moresky R, O’Neill J, et al. Emergency care in sub-Saharan Africa: Results of a consensus conference. Afr J Emerg Med. 2013 Mar 1;3(1):42–8.

Wolf L, Brysiewicz P, LoBue N, Heyns T, Bell SA, Coetzee I, et al. Developing a framework for emergency nursing practice in Africa. Afr J Emerg Med. 2012 Dec 1;2(4):174–81.

Chavula C, Pigoga JL, Kafwamfwa M, Wallis LA. Cross-sectional evaluation of emergency care capacity at public hospitals in Zambia. Emerg Med J. 2019 Oct 1;36(10):620–4.

4. *Please confirm if all data was collected in person by interviewers on site?*

*& Clarify the source of data with respect to hospital. It is not clear to me*

All data collection at each site was performed in person by teams of two to three local medical students who received training on informed consent and interviewing techniques. We have edited the section of the article on data collection for more clarity.

5. *Please include a copy of the ECAT tool, even if as an appendix, including all the signal functions for each of the 6 sentinel conditions.*

We have added a copy of our modified ECAT to our appendices

6. *In the abstract you say that you used a modification of the ECAT tool. Please provide a link to the original version of the ECAT tool.*

We cite the original ECAT in citation number 29, where a complete and original ECAT is included as appendix item A

7. *Please include a link to the full HEAT tool.*

The WHO HEAT is not publicly available, but is accessible upon request to the WHO, and have added this to our article.

See:

Pigoga JL, Joiner AP, Chowa P, Luong J, Mhlanga M, Reynolds TA, et al. Evaluating capacity at three government referral hospital emergency units in the kingdom of Eswatini using the WHO Hospital Emergency Unit Assessment Tool. BMC Emerg Med. 2020 May 6;20(1):33.

WHO Tools for strengthening emergency care systems [Internet]. [cited 2023 Jan 10]. Available from: <https://www.who.int/docs/default-source/emergencies-trauma-care/who-tools-for-strengthening-emergency-care-systems---feb-2020.pdf?sfvrsn=56f2ccf3_2>

8. *Define “infrastructural issues”.*

We now include examples of infrastructural issues on the first mention of the term, including such factors as unreliable electricity and lack of appropriate physical space to perform medical interventions.

See:

Chavula C, Pigoga JL, Kafwamfwa M, Wallis LA. Cross-sectional evaluation of emergency care capacity at public hospitals in Zambia. Emerg Med J. 2019 Oct 1;36(10):620–4.

9. *I don’t think it’s appropriate to reference an unpublished manuscript. -*

Thank you for this note. We cite a preprint of a sister study to this article to provide related epidemiological data regarding the performance of Nigerian Accident & Emergency units that complements and underpins this analysis. We believe this citation is valuable as it allows readers opportunity to assess this work in the fullest possible context. We opt not to include the full analysis performed in the cited article in this submission to limit redundancy and risk of self-plagiarism. We additionally note that citations of unpublished data are fairly standard in the health literature, with several citation conventions providing explicit means to cite unpublished work, and preprints in particular have been noted as being of particular value, for their “timeliness” and “prompt access to empirical …findings” not otherwise represented in the peer-reviewed literature, as was the case in the early stages of the COVID-19 pandemic. Here, we feel that this citation provides valuable information around the context of this work that is not otherwise available and, again, cannot be duplicated in the body of this text. We additionally feel that citation of a preprinted article offers considerable benefit over other types of unpublished data, as the cited preprinted article, though not peer-reviewed, includes detailed discussion of methodology that allows readers to approach the data it presents with an informed and critical lens. Though we feel these benefits favor the inclusion of this citation, we are willing to remove it should PLOS find that it is still not appropriate.

See:

Gianola S, Jesus TS, Bargeri S, Castellini G. Characteristics of academic publications, preprints, and registered clinical trials on the COVID-19 pandemic. PloS one. 2020 Oct 6;15(10):e0240123.

Majumder MS, Mandl KD. Early in the epidemic: impact of preprints on global discourse about COVID-19 transmissibility. The Lancet Global Health. 2020 May 1;8(5):e627-30.

10. *Did you conduct an a priori sample size calculation?*

Thank you for this note. This work was an exploratory and inductive approach to describe phenomena not yet represented in the literature, and used universal and convenience samples as opposed to random sampling methods. A priori calculations of sample size are not typical for this kind of work.

See:

Jones S, Carley S, Harrison M. An introduction to power and sample size estimation. Emergency medicine journal: EMJ. 2003 Sep;20(5):453.

11. *The Kappa value for reliability seems low*

We report kappa values to reflect interrater reliability, following best practice for qualitative research. As included in our discussion, the brevity of open-ended responses to our survey may have limited interrater reliability for initial codes and final themes, which, in both cases, was found to only be moderate. While this is the case, moderate kappa values have been viewed as acceptable elsewhere in the health literature, and particularly so outside of clinical research, where higher kappa values are important means of standardizing approaches to patients and human subjects. As this is a non-clinical study, we feel that our calculated kappa value is appropriate and important to include.

See:

Otsubo T, Tanaka K, Koda R, Shinoda J, Sano N, Tanaka S, Aoyama H, Mimura M, Kamijima K. Reliability and validity of Japanese version of the Mini‐International Neuropsychiatric Interview. Psychiatry and clinical neurosciences. 2005 Oct;59(5):517-26.

12. *Table 2 could be presented differently, perhaps using different colours for different percentages, to make it easier to read.*

We have changed the shading on this and Table 1 to make both easier to read

13. *The qualitative data presented is very light - survey text boxes are poorly equipped for qualitative analysis of any depth.*

Thank you for this note. As mentioned in our conclusion and discussion, our open-ended responses were quite brief, ranging from one to thirty words in length, and with 87% of responses under ten words long. We choose to present a sample of these responses to allow readers to critically assess our themes and the types of responses that informed them. To better aid their ability to do so, we have increased the number of participant responses presented in Table 4. We do not present more responses than are shown at present to limit redundancy.

14. *Clarify the number of tertiary hospital in Nigeria. You only indicate the method but not the number of them there. Furthermore, detail the methods used to random process.*

We now explicitly state that we randomly selected six of twenty tertiary hospitals in Nigeria, representing each of Nigeria’s six geopolitical zones.

15. *It is a little confusing if you apply some mix methods because you mention qualitative approaches, but it is not completely clear.*

Thank you for this note. This is not a mixed method study, and our focus is describing identified barriers to the provision of care at Nigerian Accident and Emergency Units. We use limited quantitative means to describe our data, but as we do not integrate our qualitative and quantitative approaches beyond this, we do not believe this work is a mixed methods study. To make this more clear, we have relabeled all mention of our “Quantitative” results section as “Descriptive Statistics”, and our “Qualitative” results section as “Open-Ended Responses”.

See:

Shorten A, Smith J. Mixed methods research: expanding the evidence base. Evidence-based nursing. 2017 Jul 1;20(3):74-5.
