# Supplementary material for: Provider-identified barriers to performance at seven Nigerian accident & emergency units: A cross-sectional study
Source: PLOS Glob Public Health. 2023 May 22;3(5):e0001909. doi: 10.1371/journal.pgph.0001909 (PMC10202278; doi:10.1371/journal.pgph.0001909)
Supplement: Attachment — Submitted filename: Barriers Review Responses 2.docx [file pgph.0001909.s003.docx]

*Reviewer #1: This mixed methods paper investigated factors impeding provision of quality emergency care in health facilities in Nigeria. This is an important topic and very well addressed by the authors. Faced with limited number of trained personnel and high burden of accidents and violence, African countries need to improve on the quality of emergency services available for patients.*

We agree with this reviewer’s perspectives, and appreciate their kind words.

*Reviewer #2: The identification of barriers to adequate healthcare access is always a relevant concern, and the accident and emergency care units play an important role in the health services’ network. On the other hand, the manuscript is clear, and the discussion presented by the authors based on the findings sounds pertinent and useful. However, some aspects need to be better calibrated in the paper:*

*1. In composing the sample of respondents with all doctors and a convenience sample of nurses, did the authors consider the implications for statistical representativeness. From whom did they want to capture the responses? Doctors and nurses in the seven healthcare units selected? In this case, wouldn't it be necessary to attribute sample weights different from one to nurses?*

Doctors, as the population among whom our survey tool (the ECAT) has been validated, were the primary respondents desired, hence the use of a universal sampling strategy to best represent this group. Nurses were also included to help triangulate data and ensure the reliability of doctors’ responses. As described in our manuscript, nurses in African contexts are critically important to the provision of emergency care, with crucial roles in the identification and triage of emergency medical conditions and patients, as well as direct patient care, making their perspectives particularly valuable for this type of analysis. The inclusion of nurses’ perspectives, then, enriches our data by providing evidence for barrier support from this vital class of healthcare provider. Weighing responses by roles would suggest that responses from different providers are of different value to our analysis, which is not the case. However, to ensure reliability, we now include results of a bivariate regression showing no significant difference in barrier endorsement by provider role.

See:

- Calvello E, Reynolds T, Hirshon JM, Buckle C, Moresky R, O’Neill J, et al. Emergency care in sub-Saharan Africa: Results of a consensus conference. Afr J Emerg Med. 2013 Mar 1;3(1):42–8.
- Wolf L, Brysiewicz P, LoBue N, Heyns T, Bell SA, Coetzee I, et al. Developing a framework for emergency nursing practice in Africa. Afr J Emerg Med. 2012 Dec 1;2(4):174–81.
  Chavula C, Pigoga JL, Kafwamfwa M, Wallis LA. Cross-sectional evaluation of emergency care capacity at public hospitals in Zambia. Emerg Med J. 2019 Oct 1;36(10):620–4.

*2. Weren’t there losses from the sample designed?*

We are unfortunately unable to report losses from our sampling strategy using available data. Despite this, our sample and analyses remain representative of Nigerian health providers and their perspectives. For quantitative analysis, a sample of 379 represents Nigeria’s physician workforce of around 25,000 medical doctors with a 5% margin of error and 95% confidence level. For qualitative analysis, convention suggests inductive analyses find thematic saturation at 30 responses. Our sample of 503 health providers, including 390 doctors, and 669 qualitative responses treated with inductive analysis, then, should adequately represent the Nigerian emergency medical physician workforce, which we expect to be substantially smaller than the broader physician workforce.

*3. Wouldn’t it be possible to list the signal functions for the sentinel conditions in a Box?*

Signal functions are shown in a copy of the modified ECAT included in our appendix. Though we feel the full list of 73 signal functions is too long to include in the body of this text, we now include sample signal functions and their associated sentinel conditions in Table 1.

*4. In Results, it does not sound appropriate the sum of individuals in Table 2 for ALL. Consequently, it would be more adequate, in the second paragraph of the section to say that a mean of 77.5% of participants reported signal functions as “Always Done”, reflecting optimal performance, while a mean of 21.1% of participants reported these as “Sometimes Done” or “Generally Not Done”, reflecting suboptimal performance.*

In the referenced portion of the manuscript, we describe signal function performance ‘across all sentinel conditions’ which we mean to indicate average values and not sums. We have now added language to make this meaning more explicit.

*5. Table 3, broken in eight fragments which are, in fact, also tables themselves, does not seem OK for me. I would prefer to have a solely table.*

Table 3 is broken into sections to display our results for barrier endorsement at all sites and across individual sites, allowing readers to consider and reference barriers in each context as fully and as conveniently as possible, as certain readers may have specific interests in specific regions of Nigeria. Merging these would yield a table too long to be easily or as conveniently read. While we have used space-saving graphical display of this data in presented media, we feel that this manuscript’s detailed discussion of our findings benefits from granular data that tables best report.

*6. Another point about Table 3 has to do with the lack of the number of respondents in each column (for all and each A&E unit), that, varies. It is a relevant information.*

At each site, the same number of respondents were surveyed with regards to each barrier, and so these numbers do not vary by sentinel condition or barrier type concerned. We have, however, added the total number of respondents at each site to the titles of all sections of Table 3.

*7. Reference 24 is cited as a complementary source of information about the work done, but it has not been published yet.*

Thank you for this note. We cite a preprint of a sister study to this article to provide related epidemiological data regarding the performance of Nigerian Accident & Emergency units that complements and underpins the analysis in the present manuscript. We believe this citation is valuable as it allows readers opportunity to assess this work in the fullest possible context. We opt to not include the full analysis of the cited article in the present manuscript to limit redundancy and risk of self-plagiarism. We additionally note that citations of unpublished data are fairly standard in the health literature, with several citation conventions providing explicit means to cite unpublished work. Preprints in particular have been noted as being of particular value, for their “timeliness” and offer of “prompt access to empirical ...findings” not otherwise represented in the peer-reviewed literature, as was the case in the early stages of the COVID-19 pandemic. Here, we feel that this citation provides valuable information around the context of this work that is not otherwise available and, again, cannot be duplicated in the body of this text. We also feel that citation of a preprinted article offers considerable benefit over other types of unpublished data, as the cited preprinted article, though not peer-reviewed, includes detailed discussion of methodology that allows readers to approach the data it presents with an informed and critical lens. Though we feel these benefits favor the inclusion of this citation, we are willing to remove it should PLOS Global Public Health find that it is still not appropriate.
See:

- Gianola S, Jesus TS, Bargeri S, Castellini G. Characteristics of academic publications, preprints, and registered clinical trials on the COVID-19 pandemic. PloS one. 2020 Oct 6;15(10):e0240123.
- Majumder MS, Mandl KD. Early in the epidemic: impact of preprints on global discourse about COVID-19 transmissibility. The Lancet Global Health. 2020 May 1;8(5):e627-30.
